# Potential for Developing Plant-Derived Candidate Vaccines and Biologics against Emerging Coronavirus Infections

**DOI:** 10.3390/pathogens10081051

**Published:** 2021-08-19

**Authors:** Balamurugan Shanmugaraj, Konlavat Siriwattananon, Ashwini Malla, Waranyoo Phoolcharoen

**Affiliations:** 1 Baiya Phytopharm Co., Ltd., Bangkok 10250, Thailand; balamurugan.s@baiyaphytopharm.com (B.S.); Ashwini.m@baiyaphytopharm.com (A.M.); 2 Research Unit for Plant-Produced Pharmaceuticals, Chulalongkorn University, Bangkok 10330, Thailand; 6176452333@student.chula.ac.th; 3 Department of Pharmacognosy and Pharmaceutical Botany, Faculty of Pharmaceutical Sciences, Chulalongkorn University, Bangkok 10330, Thailand

**Keywords:** biopharmaceuticals, coronavirus, COVID-19, molecular farming, plant expression, recombinant proteins, SARS-CoV-2

## Abstract

The emerging human coronavirus infections in the 21st century remain a major public health crisis causing worldwide impact and challenging the global health care system. The virus is circulating in several zoonotic hosts and continuously evolving, causing occasional outbreaks due to spill-over events occurring between animals and humans. Hence, the development of effective vaccines or therapeutic interventions is the current global priority in order to reduce disease severity, frequent outbreaks, and to prevent future infections. Vaccine development for newly emerging pathogens takes a long time, which hinders rapid immunization programs. The concept of plant-based pharmaceuticals can be readily applied to meet the recombinant protein demand by means of transient expression. Plants are evolved as an expression platform, and they bring a combination of unique interests in terms of rapid scalability, flexibility, and economy for industrial-scale production of effective vaccines, diagnostic reagents, and other biopharmaceuticals. Plants offer safe biologics to fulfill emergency demands, especially during pandemic situations or outbreaks caused by emerging strains. This review highlights the features of a plant expression platform for producing recombinant biopharmaceuticals to combat coronavirus infections with emphasis on COVID-19 vaccine and biologics development.

## 1. Introduction

The emergence of pathogenic human coronavirus-associated diseases has been reported in the 21st century, with severe acute respiratory syndrome coronavirus (SARS-CoV) in 2003 and Middle East respiratory syndrome coronavirus (MERS-CoV) in 2012. These viruses spread rapidly among humans worldwide, and the development of life-threatening respiratory infections caused morbidity and death in hospitalized patients, with 10% and 35% overall mortality rates, respectively [1,2]. Currently, we are witnessing the third deadly human coronavirus outbreak, which started with the reporting of the unknown pneumonia cases in Wuhan, China, in December 2019. The pneumonia infection was later named coronavirus disease 2019 (COVID-19) and was confirmed to be caused by novel coronavirus 2019 (2019-nCoV), or severe acute respiratory syndrome coronavirus (SARS-CoV-2) [3]. COVID-19 has been declared as pandemic due to its rapid human-to-human transmission around the globe in a short time period. Coronaviruses have caused three major outbreaks within two decades [4]. These alarming events show that a new coronavirus or virus variant may emerge in the near future as well. Frequent virus outbreaks cause international crisis and enormous negative impacts on public health and global economy, affecting human lifestyles and causing financial instability [5]. The development of therapeutic interventions, including effective vaccines, antibodies, diagnostic reagents, or other therapeutic proteins, is urgently needed to control the ongoing pandemic and also to avoid or prepare for any future coronavirus outbreak/pandemic [6,7,8].

Currently, few vaccines or therapeutic proteins are approved to prevent COVID-19, and many are in different stages of clinical trials. Vaccine testing requires a substantial amount of time and large quantities of drug product in order to accomplish its clinical potency [9]. Plant biopharming offers several benefits in recombinant biopharmaceutical production in terms of safety, scalability, and affordability, in comparison to traditional bacterial and mammalian expression platforms [10,11]. Plants are advantageous and can overcome the challenges associated with biopharmaceutical production, especially during an epidemic or pandemic situation, by acting as a rapid, efficient system for bulk manufacturing, thereby fulfilling the demand for biopharmaceuticals to treat infectious disease around the globe. Additionally, recombinant proteins, including vaccine candidates and monoclonal antibodies against infectious diseases, especially for Ebola, HIV, and influenza, have been produced in plants, purified, and are currently in preclinical/clinical applications [12,13,14,15,16]. Hence, plants are a fascinating platform for producing biologics or therapeutic proteins during disease outbreaks [17]. 

In this review, we have briefly discussed emerging coronavirus infections and highlighted the possibilities of using a plant-based expression system in therapeutic protein production. Further, the current status of plant-derived vaccines and immunotherapeutics against coronavirus, with emphasis on SARS-CoV-2, has been provided.

## 2. Coronaviruses 

Coronaviruses (CoVs) are enveloped viruses containing positive sense single-stranded RNA responsible for a wide range of respiratory, gastrointestinal, renal, and neurological disorders in birds, mammals, and humans [18]. CoVs belong to the order *Nidovirales* in the *Coronaviridae* family consisting of four genera: *Alphacoronavirus* (α-CoVs), *Betacoronavirus* (β-CoVs), which mainly infect mammals, and *Gammacoronavirus* (γ-CoVs), and *Deltacoronavirus* (δ-CoVs), which infect birds [19,20]. The name of coronavirus originated from the Latin word *corona*, which derives from its phenotypic characteristic crown-like appearance under the electron microscope [21]. The complete genome of CoVs typically range from 27.3 kb to 31.3 kb, consisting of 6–11 functional open reading frames encoding for structural and non-structural proteins, and other multiple accessory proteins.

Currently there are seven types of coronaviruses known to cause infections in humans, including human coronavirus 229E (HCoV-229E), HCoV-OC43, HCoV-NL63, and HCoV-HKU1. These viruses typically cause mild clinical symptoms and a self-limiting upper respiratory tract infection and may cause severe symptoms in immunodeficient patients [22,23,24]. The recent three β-CoV outbreaks, SARS-CoV, MERS-CoV, and SARS-CoV-2, are highly pathogenic zoonotic viruses that pose a serious health threat to humans. The human population witnessed the highly infectious diseases caused by these CoVs, such as severe acute respiratory syndrome (SARS) during 2002–2004, Middle East respiratory syndrome (MERS) in 2012, and most recently COVID-19 at the end of 2019 [24]. The disease was characterized with mild to severe symptoms, occasionally leading to severe respiratory syndrome, organ failure, and mortality [21,25,26]. The characteristic features of the three human pathogenic coronaviruses SARS-CoV, MERS-CoV, and SARS-CoV-2 are summarized in Table 1.

SARS-CoV and SARS-CoV-2 share the similarity in the use of angiotensin-converting enzyme 2 (ACE2) receptor for its infection [6]. SARS-CoV starts infecting the target cells by interaction with the receptor-binding domain (RBD) located in the S1 subunit of spike proteins (S) and cellular receptors resulting in the pre-fusion and viral entry. The ACE2 receptor is an important proinflammatory molecule expressing in the cells particularly in lungs, intestines, kidneys, and liver [27,28]. SARS-CoV-2 RBD is able to bind to ACE2 receptor with greater efficiency rates of 10–20-fold higher than the affinity of SARS-CoV RBD and ACE2 interaction [29].

The dipeptidyl peptidase 4 (DPP4), which is also known as CD26, is a specific cellular receptor for MERS-CoV [6]. The MERS-CoV RBD domain of S protein attaches to the DPP4 receptor on the cell surface, resulting in cleavage of S protein into S1 and S2 subunit by host proteases mediating the fusion of viral and cellular membrane, eventually releasing the viral genomic material into the host cell [30,31]. In addition, non-human primates, bats, camels, and humans are susceptible to MERS-CoV, whereas hamsters, ferrets, and mouse are not susceptible for the infection despite the presence of DPP4 [32]. DPP4 is type-II transmembrane protein presented in dimeric form on the cell surface. It is expressed on the epithelial cells in human tissues, including lung, kidney, small intestine, liver, and prostate [33]. 

## 3. Therapeutic Interventions and Vaccine Development

Therapeutic monoclonal antibodies (mAbs) have been applied for the treatment of several human diseases and have become a dominant class of pharmaceutical products developed in recent years [34]. Currently, many therapeutic mAbs have been approved by the Food and Drug Administration (FDA) and successfully used in clinical application for treating human diseases including cancers, autoimmune diseases, metabolic and infectious diseases [6,35,36,37,38]. The use of mAbs overcomes the drawbacks associated with other types of passive immunizations, particularly serum immunotherapy or intravenous immunoglobulin, in terms of specificity, safety due to low risk of human pathogen contamination, functionality, and purity [6,37]. Palivizumab was the first monoclonal antibody for infectious disease approved by the FDA in 1998 for treating the serious lung disease caused by respiratory syncytial virus in infants [39]. Since the first approval of mAb in 1998, several mAbs have been developed for infectious disease treatments. Recently, therapeutic mAbs having the potential to treat coronavirus infection have been identified. The CoV-specific mAbs targeting S or RBD demonstrate anti-viral efficacy and significantly reduce viral load by interfering the binding of RBD with its cellular receptor [38,40,41]. In addition, S-specific mAbs inhibit the viral-cellular membrane fusion in the post-fusion step, thereby blocking viral entry and infection [42]. Hence, the development of either CoV S or RBD-specific neutralizing antibodies could be an effective way for passive immune prophylaxis. Several mAbs having therapeutic potential are reported for SARS-CoV [40,43,44], MERS-CoV [43,45,46], and even for SARS-CoV-2 [47,48,49,50,51,52,53,54,55].

Nevertheless, vaccination is the most effective strategy for prevention of infectious diseases in terms of inducing long-term specific immunity, reducing severity and mortality. S protein is considered a major antigenic determinant containing the neutralizing epitopes and hence it is considered a major target for vaccine design. Specifically, the S-specific antibodies identified from recovered SARS and MERS patients showed long-lasting and immunodominant activities against viral infection [56,57]. Moreover, studies have shown that the S protein induced potent humoral and cellular immune responses in pre-clinical animal models [58,59,60,61]. Hence, S protein is considered an ideal target for vaccine development.

Different vaccine platforms have been exploited for SARS-CoV and MERS-CoV vaccine development which are either in pre-clinical development or clinical evaluations, including live-attenuated, inactivated, viral RNA, viral DNA, viral vector, and recombinant protein-based vaccines. Several vaccines targeting MERS-CoV S protein, especially RBD domain, were developed and tested in animal studies, including adenovirus vector, DNA, and protein subunit vaccines. GLS-5300, an S protein DNA-based vaccine, showed benefit in MERS-CoV protection, inducing potent neutralizing antibodies in macaques [62], and it was applied for clinical evaluation. The GLS-5300 vaccine efficiently induced immune responses up to 85% with two shots of vaccination in participants with no serious adverse effects [63]. Similarly, recombinant MERS-CoV RBD-based subunit vaccines enhance the immunogenicity profile in terms of eliciting potent neutralizing antibody and cellular immunity against MERS-CoV in animal models, cross-neutralizing human and camel MERS-CoV strains with long-lasting immunity for 6 months [64]. Recently COVID-19 vaccines were developed in different platforms and approved for emergency use including: CoronaVac (known as PiCoVacc), a whole-inactivated SARS-CoV-2 vaccine developed by Beijing-based Sinovac Biotech company [65]; inactivated SARS-CoV-2 vaccine, BBV152 (Bharat Biotech) [66]; mRNA-1273 (Massachusetts-based biotechnology company Moderna) [67]; BNT162b2 (BioNTech/Pfizer) [68]; ChAdOx1 nCoV-19 (AZD1222), a non-replicating SARS-CoV-2 viral-vectored vaccine developed by AstraZeneca [69]; and NVX-CoV2373, an S protein-based vaccine candidate developed by Novavax [70].

## 4. Plant Molecular Farming

**Figure 1:**
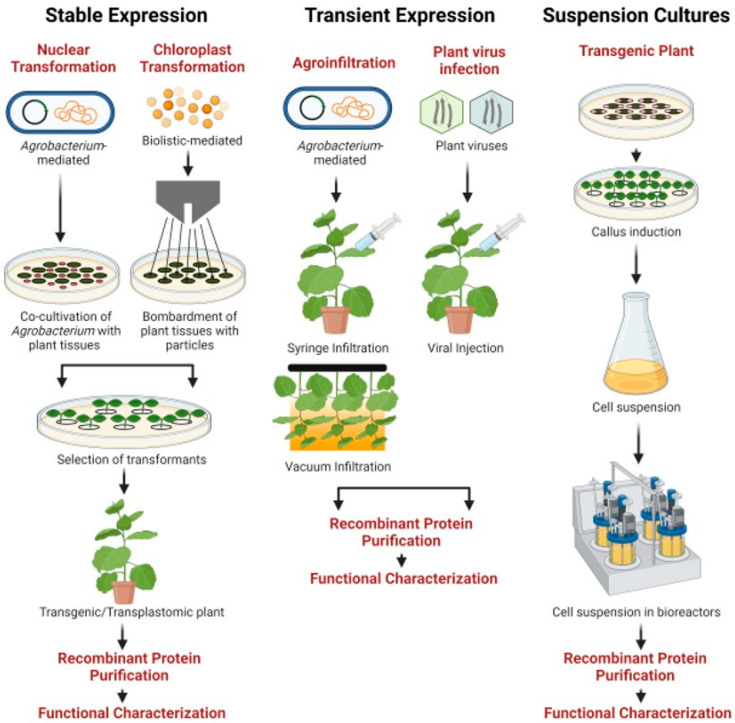
Schematic representation of available plant-based production technologies (stable, transient, and suspension cultures) for the production of recombinant vaccines and biologics [74].

Recombinant therapeutic proteins derived from biological sources, including mammalian cells, microorganisms, suspension cultures, or genetically modified organisms by employing biotechnological processes, are widely used in clinical applications, especially for the treatment and prevention of human or veterinary infections. Since the development of human insulin by using recombinant DNA technology in *E. coli* in 1982, the recombinant therapeutic protein production field has significantly grown and gained major attention [71]. The process of utilization of plants as an expression system to produce highly valuable recombinant therapeutic proteins is referred to as plant molecular farming (PMF). The concept of PMF was initially documented back in 1986 when recombinant growth hormone was produced in tobacco and sunflower plants [72]. After two decades, Elelyso (taliglucerase alfa) developed by Protalix Biotherapeutics, Israel, was approved by the U.S. FDA in 2012 [73]. Elelyso is a recombinant form of human β-glucocerebrosidase produced from carrot suspension cells. Plants have unique attractive features for protein production, including cost-effectiveness, and safety due to low risk of pathogen or toxin contamination, and are capable of performing efficient post-translational modifications essential for protein structure and functionality [10,11]. The recombinant proteins can be produced in plants via stable expression, transient expression, and plant cell-based expression (Table 2; Figure 1).

Plant transient expression is gaining interest recently due to its flexibility and rapidity in producing large quantities of recombinant biopharmaceutical proteins, including diagnostic reagents, vaccine candidates, or monoclonal antibodies to meet the demands during disease crisis or pandemic situations [81,82]. The transient expression system is widely used for production of recombinant proteins in different plant species such as *L. sativa* [83,84], *A. thaliana* [85,86], *N. tabacum* [87,88], and *N. benthamiana* [58,89,90,91,92]. Of note, *N. benthamiana* is the preferable platform for transient expression. Typically, 4–6-week-old grown plants are being used for transient expression by recombinant *Agrobacterium tumefaciens*-harboring expression cassette or viral expression vector such as tobacco mosaic virus, cowpea mosaic virus, potato virus, alfalfa mosaic virus, and plum pox virus [93]. Transient expression provides various advantages compared to other plant expression systems in terms of ease, speed, low cost, and high yield of recombinant proteins [94]. Genes of interest will be highly expressed in infiltrated plants within 3–4 days after infiltration into the plant cells [95,96,97]. For recombinant vaccine production, this strategy allows efficient production of various self-assembling viral antigens with high expression levels. The VP1 protein of foot-and-mouth disease virus was produced by using a TMV-based transient expression vector allowing the yield of approximately 0.5–1 μg/g leaf weight [98]. Similarly, hepatitis B core antigen (HBcAg) [14] and Norwalk virus (NV)-derived virus-like particles (VLPs) [99] produced using the MagnICON-based transient expression system in *N. benthamiana* plants have showed high yields of the recombinant proteins of up to 2.38 and 0.86 mg/g fresh weight (FW), respectively. Additionally, Ebola GP-based immune complex was expressed in *N. benthamiana* using a geminiviral-based transient expression vector. The maximum expression level of the antigen was obtained 4 days after agroinfiltration with the yield of approximately 50 μg antigen/g leaf mass [100]. Further, the functionality of plant-produced antigens can be confirmed by their immunogenicity profiles judged by eliciting both humoral and cell-mediated immune responses and protection from viral infection in in vivo experiments.

## 5. Plant-Based Vaccines

For almost three decades, plant expression systems have been exploited for the production of recombinant therapeutic proteins for several applications [10,101,102]. Plants have been explored for production of recombinant therapeutic proteins, especially protein-based subunit vaccines and monoclonal antibodies, to combat emerging or re-emerging diseases including COVID-19 [103]. Several proof-of-concept studies have explored the possibility of plant expression systems for the production of vaccines targeting different respiratory diseases including SARS, influenza, tuberculosis, and anthrax [15,104,105]. A plant-based vaccine against SARS utilized the stable expression of S protein (S1) in tomato and low-nicotine tobacco plants. Animal pre-clinical studies showed that the plant-derived vaccine induced an antibody response in mice [106]. Another study reported the immunogenicity of recombinant SARS-CoV N protein produced transiently in *N. benthamiana.* The tobacco produced recombinant N protein significantly induced humoral immune response after the third parental injection [107]. Further, Medicago Inc. developed the VLPs vaccine against influenza using plant-based expression technology. Influenza hemagglutinin antigens were transiently expressed in *N. benthamiana* leaves and assembled into VLPs without the viral RNA [108]. The quadrivalent seasonal influenza vaccine, which has recently completed phase III clinical evaluation, was found to be safe and immunogenic in terms of induction of humoral and T cell-mediated responses against respiratory infections caused by the influenza virus in adults [109,110]. The insights gained from previous studies can help to design and develop an effective plant-based vaccine against SARS-CoV-2, a respiratory pathogen [111]. Since the SARS-CoV-2 virus sequence was made publicly available in early 2020, significant efforts have been made by the plant molecular farming community to develop recombinant vaccines against SARS-CoV-2 (Table 3).

Medicago, a Canadian biopharmaceutical company based in Quebec City, has been involved in the development and production of a plant-based vaccine to thwart the COVID-19 infection [119]. The technology employed for vaccine manufacturing includes the synthesis of VLPs in plant cells by transient expression. VLPs without genetic material mimic the native virus structure, thus enabling the body’s immune system to induce a robust immune response. The same technology has been utilized for the production of a VLP vaccine candidate against influenza, which demonstrated immunogenicity and efficacy in trials. The COVID-19 VLP (Co-VLP) developed at Medicago uses full-length spike protein from SARS-CoV-2 viral genome that trimerize and assemble into VLP inside plant cells. The plant synthesized Co-VLPs were formulated with two adjuvants: CpG 1018: Dynavax, and AS03: GSK, and their safety, immunogenicity, and protection was evaluated in non-human primates. It was found that the vaccine candidates adjuvanted with AS03 elicited a more potent immune response than the CpG 1018-adjuvanted formulations. Further, no adverse reactions related to vaccine-associated enhanced disease were observed. This candidate vaccine was evaluated in the phase I clinical studies in healthy humans carried out on 180 healthy individuals in the 18–55 years age group. Two doses of three different strengths: 3.75 µg, 7.5 µg, and 15 µg, adjuvanted independently with AS03 and CpG 1018, were administered to the individuals at 21 days apart, and the safety, immunogenicity, and efficacy was assessed after 42 days. A robust immune response was observed with ten-fold higher titers in the groups administered with Co-VLP adjuvanted with AS03 in addition to spike protein-specific interferon-γ and interleukin-4 cellular responses. Based on these results from phase I clinical trials and nonclinical trials, the study is being further progressed globally, covering Canada, USA, and other countries, and is currently in a phase III trial administering CoVLP+AS03 at a dose level of 3.75 µg [120,121].

Kentucky BioProcessing (KBP) based in Owensboro, Kentucky, is also competing to commercialize its innovative fast-growing plant-based COVID-19 vaccine KBP-201, which utilizes *N. benthamiana* as an expression host [119]. KBP-201 adjuvanted with CpG oligonucleotide is currently in phase I/II clinical trials in United States, with the study designed to test 180 healthy volunteers from two age ranges: 18–49 years and 50–70 years, with low and high doses of vaccine (ClinicalTrials.gov Identifier: NCT04473690).

iBio, located in Bryan, Texas, is developing plant-based candidate vaccines IBIO-200, a VLP-based vaccine, IBIO-201, a SARS-CoV-2 spike-based subunit vaccine combined with LicKM™ booster molecule, and IBIO-202, a subunit vaccine candidate targeting the nucleocapsid protein of SARS-CoV-2. Their manufacturing process focused on coupling their FastPharming and LicKM technologies using VLPs and subunit vaccine platforms to produce the COVID-19 vaccine [119]. The emergence of mutated coronavirus strains has raised concerns and diverted their focus to developing a second-generation vaccine (IBIO-202) with broader protection that uses the highly conserved regions of SARS-CoV-2 nucleocapsid protein containing immunogenic epitopes with the intention that the newer variants may be less prone to escape vaccine protection [122,123,124,125]. Currently, the IBIO-201 vaccine manufactured in tobacco plants has completed preclinical studies with no adverse effects at low and high doses in mice, whereas the preclinical trial of IBIO-202 has been completed in July 2021 [116,117].

Baiya Phytopharm, a start-up company in Thailand, is using the BaiyaPharming™ protein expression platform to develop a subunit-based vaccine against SARS-CoV-2 in *N. benthamiana.* Six vaccine candidates were tested for their efficacy and based on the results one candidate was chosen (Baiya SARS-CoV-2 Vax 1), which showed better immunogenicity in mice and monkeys. Further safety and efficacy studies were carried out with the intention of starting the phase I clinical trials from September 2021 (ClinicalTrials.gov Identifier: NCT04953078) [126].

## 6. Plant-Derived Antibodies and Diagnostic Reagents

In addition to vaccine antigens, the production of mAbs in plants is well known, and hence the plants can also be employed for the rapid production of mAbs to fight against viral infection. Plants are capable of producing fully assembled functional mAbs for human and veterinary applications. Antibody-mediated passive immunization can confer immediate protection against infection caused by contagious pathogens. Antibodies against several infectious agents have been successfully produced in plants. A humanized mAb against West Nile virus was produced using MagnICON technology to accumulate high levels of protein and showed protective efficacy in animal challenge experiments [127]. The anti-HIV 2G12 mAb was produced up to 325 μg/g FW when expressed in tobacco plants by using a CPMV based vector [128]. Plant-produced chimeric D5 antibody against Enterovirus 71 was expressed with highest expression level on day 6 post-agroinfiltration with the yield of 50 μg/g FW and exhibited the protective efficacy in mice challenge studies [90]. In parallel, a geminiviral expression vector used for producing anti-human PD1 antibody by co-infiltration of heavy chain and light chain in *N. benthamiana* yielded up to 140 μg of mAb/g FW and its functional/biological activities were confirmed in in vitro studies [91]. Of note, Mapp Biopharmaceutical Inc. (San Diego, MO, USA) has developed a plant-derived mAb cocktail, named ZMapp, which combines three chimeric mAbs, c13C6, c2G4, and c4G7, and was produced by transient expression in tobacco plants, in order to be used as a passive immunotherapy for Ebola-infected patients [13].

Recently, anti-SARS-CoV/CoV-2 mAbs were also expressed as a proof-of-concept to show the efficiency of plant expression systems in the production of CoV mAbs for passive immunotherapy. Our group has reported the production of human anti-SARS-CoV-2 mAbs B38 and H4 in *N. benthamiana* using geminiviral vector. The transient co-expression of light and heavy chain genes of antibodies in plant leaves accumulates 4 to 35 μg of mAb/g FW. Both plant-produced mAbs retained antigen binding specificity and exhibited neutralizing activity against SARS-CoV-2 in vitro [47]. Diego-Martin and colleagues reported the expression of six anti-SARS-CoV-2 antibodies in *N. benthamiana* with the expression level ranging from 73 to 192 μg/g FW [114]. Furthermore, SARS-CoV-2 antigens expressed in plants have also been utilized as diagnostic reagents to develop rapid test kits for SARS-CoV-2 infection (Table 4). A group in South Africa demonstrated that the recombinant plant-produced S1 and RBD of SARS-CoV-2 enable the detection of SARS-CoV-2-specific antibodies in patient sera who had tested positive by PCR [129]. Baiya rapid COVID-19 IgM/IgG test kit based on a lateral-flow immunoassay strip was developed by Baiya Phytopharm in Thailand using recombinant RBD of SARS-CoV-2 produced from plants. A total of 51 confirmed COVID-19 serum samples were tested using the strip, and the sensitivity and specificity of the kit was reported to be 94.1% and 98%, respectively [130]. The list of mAbs and diagnostic reagents produced in plants are outlined in Table 4.

## 7. Prospective View

Significant progress has been made in the development of plant-derived COVID-19 vaccines by applying the existing knowledge and proof-of-concept evidence on other plant-derived candidates. SARS-CoV-2 vaccine candidates expressed in plants have been reported with promising results. In a recent report, a fusion protein comprised of a sequence of RBD of SARS-CoV-2 and Fc region of immunoglobulin has been fused and expressed in *N. benthamiana* plants by transient expression. The plant-derived RBD-Fc fusion protein was reported to induce broad humoral and cellular responses when intramuscularly injected in mice and cynomolgus macaques. The importance of adjuvants in enhancing the immune response of plant-derived subunit vaccines against SARS-CoV-2 was also demonstrated [58,134]. Such subunit vaccine strategies can be considered further for safety and efficacy studies. In particular, glycosylation plays a significant role in antigenic properties of vaccine antigens. Although plants can perform post-translational modifications somewhat similar to human and mammalian cells, the difference in glycosylation patterns in plant-derived proteins can be overcome by glycoengineering in plants to obtain humanized proteins [135], which would serve as a tool to develop SARS-CoV-2 vaccines in plants. Plant-derived vaccines might serve as a cost-effective platform to produce COVID-19 vaccines, which in turn reduces the vaccination costs and feasibility to attain large-scale immunization programs. The assessment of SARS-CoV-2 VLP and subunit vaccines produced in plants may substantially contribute to advancing the field further, however, it is important to explore the possibilities of developing a plant-vaccine to induce broad immunoprotection against emerging SARS-CoV-2 variants as well.

In conclusion, researchers across the world are making significant efforts by utilizing all the available platforms to develop an effective, safe vaccine to control COVID-19. With the advent of transient production technology, the plant expression system is considered as a viable approach, and it has also been gaining interest among the pharmaceutical companies in recent years for recombinant biopharmaceutical production, due to its ability to produce large doses of vaccine antigens or therapeutic proteins in a short time. While several scientific teams in the plant molecular farming community are working on the development of plant-derived vaccines, one must accept the fact that the regulatory pathway for approval of new vaccines is complex and time-consuming, and the advantages of the plant expression platform can be realized only after overcoming regulatory hurdles. Harmonizing the regulatory procedures for plant-derived products (plant-specific regulation), both at national and international levels, is essential for reducing the timeframe of plant biologics from bench to market. Further, the plant-derived products must meet the quality standards and all applicable stringent current Good Manufacturing Practice guidelines devised for biological products. Currently, few plant-produced vaccine candidates against SARS-CoV-2 have reached preclinical and clinical trials. Although the progress of commercialization of plant-derived vaccines has been slow, the promising results in clinical trials of plant-derived influenza and COVID-19 vaccine in recent years encourage the confidence that we can expect the commercialization of plant-derived vaccine in this upcoming decade.

## Figures and Tables

**Table 1:** Comparative analysis of biological features of SARS, MERS, and COVID-19 (As of 13 August 2021).

Disease	SARS	MERS	COVID-19
**Epidemiology**			
First reported case	February, 2003	June, 2012	December, 2019
Country of diagnosis	China	Saudi Arabia	China
Initial name	Novel coronavirus	Human coronavirus-Erasmus Medical Center (HCoV-EMC)	2019 novel coronavirus (2019-nCoV)
Disease	SARS	MERS	COVID-19
Current status	Disappeared	Active	Active (Ongoing)
Pandemic status	No	No	Yes
Highly affected countries	China, Hong Kong, Taiwan, Singapore, Canada	Saudi Arabia, United Arab Emirates, South Korea	USA, India, Brazil, Russia, France
Total number of cases	8096 confirmed cases (24 July 2015)	2574 confirmed cases (11 March 2021)	203,944,144 confirmed cases (12 August 2021)
Number of countries affected	29	27	>200
Fatality rate	~10%	~35%	~2.2
**Genome organization**			
Order	*Nidovirales*	*Nidovirales*	*Nidovirales*
Family	*Coronaviridae*	*Coronaviridae*	*Coronaviridae*
Sub-family	*Orthocoronavirinae*	*Orthocoronavirinae*	*Orthocoronavirinae*
Genus	*Betacoronavirus*	*Betacoronavirus*	*Betacoronavirus*
Sub-genus	*Sarbecovirus*	*Merbecovirus*	*Sarbecovirus*
Lineage	B	C	B
Genome type	ssRNA (+)	ssRNA (+)	ssRNA (+)
Genome size	29.7 kb	30.1 kb	29.9 kb
Structural proteins	Spike protein (S), Membrane protein (M), Nucleocapsid protein (N), Envelope protein (E)	Spike protein (S), Membrane protein (M), Nucleocapsid protein (N), Envelope protein (E)	Spike protein (S), Membrane protein (M), Nucleocapsid protein (N), Envelope protein (E)
Natural host	Bats	Bats	Bats
Intermittent host	Palm civet cats	Dromedary camels	Pangolins
Viral protein that binds to receptor	Spike protein, especially receptor-binding motif (RBM)	Spike protein, especially receptor-binding motif (RBM)	Spike protein, especially receptor-binding motif (RBM)
Functional receptor	Human angiotensin-converting enzyme 2 (ACE2)	Human dipeptidyl peptidase 4 (DPP4 or CD26)	Human angiotensin-converting enzyme 2 (ACE2)
Receptor localized organ	Lung, intestine, kidneys, heart, liver, and testicles	Brain, heart, lung, kidney, spleen, intestine, and liver	Lungs, intestines, kidneys, heart, liver, and testicles
Virus variants	N/A	N/A	Alpha, Beta, Delta, Gamma, Lota, Kappa, Eta, Lambda
**Symptoms**			
Flu-like Symptoms	Fever, myalgia, headache, malaise, dyspnea, chills, and rigors	Fever and cough, chills, rigor, rhinorrhea, myalgia, and fatigue	Fever, chills, coughing, breathlessness, fatigue, muscle ache, headache, sore throat, congestion, running nose, loss of smell, and loss of taste
Severe Symptoms	Hypoxemia, severe respiratory illness, low white blood cell counts, and low platelet counts	Acute respiratory distress syndrome (ARDS), septic shock, multi organ failures, and respiratory failure	Common complications consisting of pneumonia, acute respiratory syndrome, liver injury, myocarditis, acute kidney injury, neurological complication, cardiopulmonary failure, acute cerebrovascular disease, and shock
Gastrointestinal Symptoms	N/A	Nausea, vomiting, diarrhea, and abdominal pain	Nausea, vomiting, diarrhea
Asymptomatic	No	Yes	Yes
Latency Period	2–7 days after viral exposure but may be as long as 10 days	5–12 days after viral exposure	2–12 days after viral exposure
Mode of transmission	Human-to-human	Human-to-human	Human-to-human
**Diagnosis and treatment**			
Diagnostic procedure	Molecular tests by RT-PCR; other laboratory and radiographic findings	Molecular tests by RT-PCR; other laboratory and radiographic findings	Molecular tests by RT-PCR; other laboratory and radiographic findings
Number of vaccines in clinical phase	2	3	110
Approved vaccines	N/A	N/A	21 vaccines approved by at least one country. 7 vaccines approved for use by WHO

N/A: not available.

**Table 2:** Available expression strategies for the production of biopharmaceutical proteins in plants.

Expression Strategy	Advantages	Disadvantages	Examples
Stable expression	Scalability Can apply in different plant crops Transgenic seeds can be stored for a long time	Time-consuming, tedious, and labor intensive Incorporation of transgene in plant genome and variability in transgene expression Risk of gene silencing/position effect Transgene contamination Random gene integration	Anti-HIV 2G12 IgG in transgenic tobacco [75] Anti-rabies E559 IgG in in transgenic tobacco [76]
Transient expression	Gene of interest does not integrate with plant genome Ease of manipulation Simple technology, rapid expression High yield Efficient and timesaving process Can utilize for rapid recombinant protein production during emergency situations	Protein yield may not be consistent and varies among individual plants Chances of endotoxin contamination from *Agrobacterium* origin	Anti-Ebola antibody cocktail in tobacco [36] Influenza virus (H5N1)-based VLP vaccine in tobacco [77] Anti-RANKL mAb in tobacco [78]
Suspension cells	Sterile production environment Chemically defined media lacking animal components Compatible with regulatory guidelines Simple downstream processing Scalability	Complexity in large-scale production Genetic instability and reduced productivity over long time periods High cost of cultivation	Glucocerebrosidase enzyme in carrot cells [73] Human β-1,4-galactosyltransferase in BY-2 tobacco cell lines [79] Alpha-galactosidase-A (Fabrazyme) in tobacco cell lines [80]

**Table 3:** List of vaccine candidates against SARS-CoV/CoV-2 produced in plants (As of 13 August 2021).

Vaccine Antigen	Plant Host	Formulation and Route	Immunogenicity	Status	Reference
SARS-CoV S1 protein	Stable expression in tomato	GI with 2-week intervals for 3 doses	Significantly increased titers of SARS-CoV-specific antibodies after immunization in mice	Pre-clinical study	[106]
SARS-CoV nucleocapsid protein	Transient expression in *N. benthamiana*	Formulated with Freund’s adjuvant and IP with at 2-week intervals for four doses	Able to induce humoral immunity as well as SARS-CoV-2 cytokine-producing cells in mice	Pre-clinical study	[107]
SARS-CoV S1-GFP fusion protein	Transient expression in *N. benthamiana*	N/A	N/A	Research	[112]
Recombinant SARS-CoV N and M protein	Transient expression in *N. benthamiana*	N/A	N/A	Research	[113]
Recombinant SARS-CoV-2 N protein	Transient expression in *N. benthamiana*	N/A	N/A	Research	[114]
Recombinant SARS-CoV-2 RBD protein	Transient expression in *N. benthamiana*	N/A	N/A	Research	[114]
KBP-201 COVID-19 vaccine: SARS-CoV-2 RBD-based vaccine developed by Kentucky BioProcessing, Inc. (Owensboro, KY, USA)	Transient expression in *N. benthamiana*	Formulated with CpG adjuvant and IM injection on day 1 and 22	Able to induce positive SARS-CoV-2-specific immunity in pre-clinical trials	Phase I/II ClinicalTrials.gov Identifier: NCT04473690	[115]
IBIO-200 vaccine: SARS-CoV-2 VLP-based vaccine developed by iBio, Inc. (Bryan, TX, USA)	Transient expression in *N. benthamiana*	IM injection on day 1 and 21	Able to stimulate specific immune responses and neutralizing antibody against SARS-CoV-2 in mice	Pre-clinical study	[116]
IBIO-201 vaccine: SARS-CoV-2 Spike-based sub-unit vaccine developed by iBio, Inc. (Bryan, TX, USA)	Transient expression in *N. benthamiana*	Formulated with LicKM^TM^ adjuvant and IM injection on day 1 and 21	Able to stimulate specific immune responses and neutralizing activities against SARS-CoV-2 in mice more than IBIO-200	Pre-clinical study	[116]
IBIO-202 vaccine: SARS-CoV-2 nucleocapsid protein-based sub-unit vaccine developed by iBio, Inc. (Bryan, TX, USA)	Transient expression in *N. benthamiana*	N/A	Able to induce robust, antigen-specific, memory T cell response	Pre-clinical study	[117]
SARS-CoV-2 VLP-based vaccine developed by Medicago Inc. (Quebec City, QC, Canada, Canada)	Transient expression in *N. benthamiana*	IM injection given 21 days apart	Able to induce antibody responses in the volunteers with two doses of immunization	Phase II/III ClinicalTrials.gov Identifier: NCT04636697	[118]
Baiya SARS-CoV-2 Vax 1 subunit vaccine developed by Baiya Phytopharm Co., Ltd. (Bangkok, Thailand)	Transient expression in *N. benthamiana*	Formulated with alum adjuvant and IM injection on day 1 and 21	Able to induce antigen-specific IgG and neutralizing responses as well as cellular immunity in mice and non-human primates	Pre-clinical study	[58]

N/A: not available; GI: gastric intubation; IM: intramuscular immunization.

**Table 4:** List of plant-produced mAbs and diagnostic reagents developed for the treatment and diagnosis of coronavirus infection (As of 13 August 2021).

Product	Expression/Plant Host	Attractive Features	Status	Reference
**Plant-produced mAbs**				
Anti-SARS-CoV-2 B38 and H4 mAb	Transient expression in *N. benthamiana*	Specific binding to RBD domain on S1 subunit of SARS-CoV-2 and exhibited neutralizing activity against viral infection in vitro	Research	[47]
Anti-SARS-CoV CR3022 mAb	Transient expression in *N. benthamiana*	Specific binding to RBD domain on S1 subunit of SARS-CoV and SARS-CoV-2	Research	[81]
Anti-SARS-CoV-2 sybody3 VHH-Fc IgG1	Transient expression in *N. benthamiana*	Specific binding to RBD domain on S1 subunit of SARS-CoV-2	Research	[114]
Anti-SARS-CoV-2 sybody17 VHH-Fc IgG1	Transient expression in *N. benthamiana*	Specific binding to RBD domain on S1 subunit of SARS-CoV-2	Research	[114]
Anti-SARS-CoV/CoV-2 nanobody72 VHH-Fc IgG1	Transient expression in *N. benthamiana*	Specific binding to RBD domain on S1 subunit of SARS-CoV-2	Research	[114]
Anti-SARS-CoV nucleocapsid (N) CR3009-scFv	Transient expression in *N. benthamiana*	Specific binding to N protein of SARS-CoV and SARS-CoV-2	Research	[114]
Anti-SARS-CoV nucleocapsid (N) CR3018-scFv	Transient expression in *N. benthamiana*	Specific binding to N protein of SARS-CoV and SARS-CoV-2	Research	[114]
**Other therapeutic protein**				
ACE2-Fc fusion protein developed by Baiya Phytopharm Co., Ltd. (Bangkok, Thailand)	Transient expression in *N. benthamiana*	Blocking and neutralizing RBD domain on S1 subunit of SARS-CoV-2	Research	[89]
ACE2-Fc fusion protein developed by iBio, Inc. (Bryan, TX, USA)	Transient expression in *N. benthamiana*	ACE2-Fc blocks SARS-CoV-2 virus from infecting Vero E6 cells	Research	[131]
**Research and diagnostics reagents**				
SARS-CoV-2 RBD-based ELISA test kit developed by Diamante, Italian biotech company	Transient expression in *N. benthamiana*	Used in ELISA for the detection of serum antibody in COVID-19 convalescent patients	Production	[132]
Baiya rapid COVID-19 IgM/IgG test kit developed by Baiya Phytopharm Co., Ltd. (Bangkok, Thailand)	Transient expression in *N. benthamiana*	Used as lateral-flow immunoassay strip reagents for the detection of IgM/IgG antibodies in human sera	Production	[130]
Recombinant SARS-CoV-2 nucleocapsid protein developed by Leaf Expression System (Norwich, UK)	Transient expression in *N. benthamiana*	Used as an antigen diagnostic reagent	Production	[133]

N/A: not available.

## Data Availability

Not applicable.

## Abbreviations

ACE2	Angiotensin-Converting Enzyme 2
cGMP	Current Good Manufacturing Practices
CoV	Coronavirus
COVID-19	Coronavirus Disease 2019
ELISA	Enzyme Linked Immunosorbent Assay
DPP4	Di-Peptidyl Peptidase 4
FDA	Food and Drug Administration
FW	Fresh Weight
IM	Intramuscular
IP	Intraperitoneal
mAbs	Monoclonal Antibodies
MERS	Middle East Respiratory Syndrome
MERS-CoV	Middle East Respiratory Syndrome Coronavirus
*N. benthamiana*	*Nicotiana benthamiana*
PMF	Plant Molecular Farming
RBD	Receptor-Binding Domain
S protein	Spike protein
SARS	Severe Acute Respiratory Syndrome
SARS-CoV	Severe Acute Respiratory Syndrome Coronavirus
SARS-CoV-2	Severe Acute Respiratory Syndrome Coronavirus - 2
VLPs	Virus-Like Particles
α-coronavirus	Alphacoronavirus
Β-coronavirus	Betacoronavirus
δ-coronavirus	Deltacoronavirus
ϒ-coronavirus	Gammacoronavirus
2019-nCoV	Novel Coronavirus 2019

## References

[1] World Health Organization MERS Situation Update September 2019. https://applications.emro.who.int/docs/EMROPub-MERS-SEP-2019-EN.pdf?ua=1&ua=1.

[2] World Health Organization Summary of Probable SARS Cases with Onset of Illness from 1 November 2002 to 31 July 2003. https://www.who.int/publications/m/item/summary-of-probable-sars-cases-with-onset-of-illness-from-1-november-2002-to-31-july-2003.

[3] World Health Organization Coronavirus Disease (COVID-19) Outbreak. https://www.who.int/emergencies/diseases/novel-coronavirus-2019.

[4] Chan J.F.-W., Yuan S., Kok K.-H., To K.K.-W., Chu H., Yang J., Xing F., Liu J., Yip C.C.-Y., Poon R.W.-S. (2020). A familial cluster of pneumonia associated with the 2019 novel coronavirus indicating person-to-person transmission: A study of a family cluster. Lancet.

[5] Gralinski L.E., Baric R.S. (2015). Molecular pathology of emerging coronavirus infections. J. Pathol..

[6] Shanmugaraj B., Siriwattananon K., Wangkanont K., Phoolcharoen W. (2020). Perspectives on monoclonal antibody therapy as potential therapeutic intervention for Coronavirus disease-19 (COVID-19). Asian Pac. J. Allergy Immunol..

[7] Zhu X., Liu Q., Du L., Lu L., Jiang S. (2013). Receptor-binding domain as a target for developing SARS vaccines. J. Thorac. Dis..

[8] Malla A., Shanmugaraj B., Ramalingam S. (2020). Severe Acute Respiratory Syndrome Coronavirus-2 (SARS-CoV-2): An Emerging Zoonotic Respiratory Pathogen in Humans. J. Pure Appl. Microbiol..

[9] Lagassé H.A.D., Alexaki A., Simhadri V.L., Katagiri N.H., Jankowski W., Sauna Z.E., Kimchi-Sarfaty C. (2017). Recent advances in (therapeutic protein) drug development. F1000Research.

[10] Shanmugaraj B., Bulaon C.J.I., Phoolcharoen W. (2020). Plant molecular farming: A viable platform for recombinant biopharmaceutical production. Plants.

[11] Burnett M.J.B., Burnett A.C. (2019). Therapeutic recombinant protein production in plants: Challenges and opportunities. Plants People Planet.

[12] Park K.Y., Wi S.J. (2016). Potential of plants to produce recombinant protein products. J. Plant Biol..

[13] Donini M., Marusic C. (2019). Current state-of-the-art in plant-based antibody production systems. Biotechnol. Lett..

[14] Huang Z., Santi L., LePore K., Kilbourne J., Arntzen C.J., Mason H.S. (2006). Rapid, high-level production of hepatitis B core antigen in plant leaf and its immunogenicity in mice. Vaccine.

[15] D’Aoust M.-A., Couture M.M.-J., Charland N., Trépanier S., Landry N., Ors F., Vézina L.-P. (2010). The production of hemagglutinin-based virus-like particles in plants: A rapid, efficient and safe response to pandemic influenza. Plant Biotechnol. J..

[16] Takeyama N., Kiyono H., Yuki Y. (2015). Plant-based vaccines for animals and humans: Recent advances in technology and clinical trials. Ther. Adv. Vaccines.

[17] Shanmugaraj B., Malla A., Phoolcharoen W. (2020). Emergence of Novel Coronavirus 2019-nCoV: Need for Rapid Vaccine and Biologics Development. Pathogens.

[18] Masters P.S. (2006). The Molecular Biology of Coronaviruses. Adv. Virus Res..

[19] Fung S.-Y., Yuen K.-S., Ye Z.-W., Chan C.-P., Jin D.-Y. (2020). A tug-of-war between severe acute respiratory syndrome coronavirus 2 and host antiviral defence: Lessons from other pathogenic viruses. Emerg. Microbes Infect..

[20] Li F. (2016). Structure, function, and evolution of coronavirus spike proteins. Annu. Rev. Virol..

[21] Li Y.-D., Chi W.-Y., Su J.-H., Ferrall L., Hung C.-F., Wu T.-C. (2020). Coronavirus vaccine development: From SARS and MERS to COVID-19. J. Biomed. Sci..

[22] Cui J., Li F., Shi Z.-L. (2019). Origin and evolution of pathogenic coronaviruses. Nat. Rev. Microbiol..

[23] Zhou P., Yang X.-L., Wang X.-G., Hu B., Zhang L., Zhang W., Si H.-R., Zhu Y., Li B., Huang C.-L. (2020). A pneumonia outbreak associated with a new coronavirus of probable bat origin. Nature.

[24] Lauxman M.A., Santucci N.E., Autrán-Gómez A.M. (2020). The SARS-CoV-2 Coronavirus and the COVID-19 Outbreak. Int. Braz. J. Urol..

[25] de Wit E., van Doremalen N., Falzarano D., Munster V.J. (2016). SARS and MERS: Recent insights into emerging coronaviruses. Nat. Rev. Microbiol..

[26] Zhu N., Zhang D., Wang W., Li X., Yang B., Song J., Zhao X., Huang B., Shi W., Lu R. (2020). A novel coronavirus from patients with pneumonia in China, 2019. N. Engl. J. Med..

[27] Kuba K., Imai Y., Penninger J.M. (2006). Angiotensin-converting enzyme 2 in lung diseases. Curr. Opin. Pharmacol..

[28] Hamming I., Timens W., Bulthuis M.L.C., Lely A.T., Navis G.J., van Goor H. (2004). Tissue distribution of ACE2 protein, the functional receptor for SARS coronavirus. A first step in understanding SARS pathogenesis. J. Pathol..

[29] Nguyen H.L., Lan P.D., Thai N.Q., Nissley D.A., O’Brien E.P., Li M.S. (2020). Does SARS-CoV-2 bind to human ACE2 more strongly than does SARS-CoV?. Phys. Chem. B.

[30] Rasmussen S.A., Watson A.K., Swerdlow D.L. (2016). Middle East respiratory syndrome (MERS). Microbiol. Spectr..

[31] Mou H., Raj V.S., van Kuppeveld F.J.M., Rottier P.J.M., Haagmans B.L., Bosch B.J. (2013). The receptor binding domain of the new Middle East respiratory syndrome coronavirus maps to a 231-residue region in the spike protein that efficiently elicits neutralizing antibodies. J. Virol..

[32] van Doremalen N., Miazgowicz K.L., Milne-Price S., Bushmaker T., Robertson S., Scott D., Kinne J., McLellan J.S., Zhu J., Munster V.J. (2014). Host species restriction of Middle East respiratory syndrome coronavirus through its receptor, dipeptidyl peptidase 4. J. Virol..

[33] Meyerholz D.K., Lambertz A.M., McCray P.B. (2016). Dipeptidyl Peptidase 4 Distribution in the Human Respiratory Tract: Implications for the Middle East Respiratory Syndrome. Am. J. Pathol..

[34] Ning L., Abagna H.B., Jiang Q., Liu S., Huang J. (2021). Development and application of therapeutic antibodies against COVID-19. Int. J. Biol. Sci..

[35] Lu R.-M., Hwang Y.-C., Liu I.-J., Lee C.-C., Tsai H.-Z., Li H.-J., Wu H.-C. (2020). Development of therapeutic antibodies for the treatment of diseases. J. Biomed. Sci..

[36] Davey R.T., Dodd L., Proschan M.A., Neaton J., Nordwall J.N., Koopmeiners J.S., Beigel J., Tierney J., Lane H.C., Fauci A.S. (2016). A randomized, controlled trial of ZMapp for ebola virus infection. N. Engl. J. Med..

[37] Bayry J., Lacroix-Desmazes S., Kazatchkine M.D., Kaveri S.V. (2007). Monoclonal antibody and intravenous immunoglobulin therapy for rheumatic diseases: Rationale and mechanisms of action. Nat. Clin. Pract. Rheumatol..

[38] Tuccori M., Ferraro S., Convertino I., Cappello E., Valdiserra G., Blandizzi C., Maggi F., Focosi D. (2020). Anti-SARS-CoV-2 neutralizing monoclonal antibodies: Clinical pipeline. MAbs.

[39] The IMpact-RSV Study Group (1998). Palivizumab, a humanized respiratory syncytial virus monoclonal antibody, reduces hospitalization from respiratory syncytial virus infection in high-risk infants. Pediatrics.

[40] Sui J., Li W., Murakami A., Tamin A., Matthews L.J., Wong S.K., Moore M.J., Tallarico A.S.C., Olurinde M., Choe H. (2004). Potent neutralization of severe acute respiratory syndrome (SARS) coronavirus by a human mAb to S1 protein that blocks receptor association. Proc. Natl. Acad. Sci. USA.

[41] Jiang L., Wang N., Zuo T., Shi X., Poon K.-M.V., Wu Y., Gao F., Li D., Wang R., Guo J. (2014). Potent neutralization of MERS-CoV by human neutralizing monoclonal antibodies to the viral spike glycoprotein. Sci. Transl. Med..

[42] Lip K.-M., Shen S., Yang X., Keng C.-T., Zhang A., Oh H.-L.J., Li Z.-H., Hwang L.-A., Chou C.-F., Fielding B.C. (2006). Monoclonal antibodies targeting the HR2 domain and the region immediately upstream of the HR2 of the S protein neutralize in vitro infection of Severe acute respiratory syndrome coronavirus. J. Virol..

[43] Sui J., Li W., Roberts A., Matthews L.J., Murakami A., Vogel L., Wong S.K., Subbarao K., Farzan M., Marasco W.A. (2005). Evaluation of human monoclonal antibody 80R for immunoprophylaxis of severe acute respiratory syndrome by an animal study, epitope mapping, and analysis of spike variants. J. Virol..

[44] Roberts A., Vogel L., Guarner J., Hayes N., Murphy B., Zaki S., Subbarao K. (2005). Severe acute respiratory syndrome coronavirus infection of golden Syrian hamsters. J. Virol..

[45] Yu X., Zhang S., Jiang L., Cui Y., Li D., Wang D., Wang N., Fu L., Shi X., Li Z. (2015). Structural basis for the neutralization of MERS-CoV by a human monoclonal antibody MERS-27. Sci. Rep..

[46] Houser K.V., Gretebeck L., Ying T., Wang Y., Vogel L., Lamirande E.W., Bock K.W., Moore I.N., Dimitrov D.S., Subbarao K. (2016). Prophylaxis with a Middle East respiratory syndrome coronavirus (MERS-CoV)-specific human monoclonal antibody protects rabbits from MERS-CoV infection. J. Infect. Dis..

[47] Shanmugaraj B., Rattanapisit K., Manopwisedjaroen S., Thitithanyanont A., Phoolcharoen W. (2020). Monoclonal antibodies B38 and H4 produced in *Nicotiana benthamiana* neutralize SARS-CoV-2 in vitro. Front. Plant Sci..

[48] Baum A., Ajithdoss D., Copin R., Zhou A., Lanza K., Negron N., Ni M., Wei Y., Mohammadi K., Musser B. (2020). REGN-COV2 antibodies prevent and treat SARS-CoV-2 infection in rhesus macaques and hamsters. Science.

[49] Shi R., Shan C., Duan X., Chen Z., Liu P., Song J., Song T., Bi X., Han C., Wu L. (2020). A human neutralizing antibody targets the receptor-binding site of SARS-CoV-2. Nature.

[50] Zost S.J., Gilchuk P., Chen R.E., Case J.B., Reidy J.X., Trivette A., Nargi R.S., Sutton R.E., Suryadevara N., Chen E.C. (2020). Rapid isolation and profiling of a diverse panel of human monoclonal antibodies targeting the SARS-CoV-2 spike protein. Nat. Med..

[51] Du L., He Y., Zhou Y., Liu S., Zheng B.-J., Jiang S. (2009). The spike protein of SARS-CoV--a target for vaccine and therapeutic development. Nat. Rev. Microbiol..

[52] Wang L., Shi W., Chappell J.D., Joyce M.G., Zhang Y., Kanekiyo M., Becker M.M., van Doremalen N., Fischer R., Wang N. (2018). Importance of neutralizing monoclonal antibodies targeting multiple antigenic sites on the middle east respiratory syndrome coronavirus spike glycoprotein to avoid neutralization escape. J. Virol..

[53] Wu Y., Wang F., Shen C., Peng W., Li D., Zhao C., Li Z., Li S., Bi Y., Yang Y. (2020). A noncompeting pair of human neutralizing antibodies block COVID-19 virus binding to its receptor ACE2. Science.

[54] Wang C., Li W., Drabek D., Okba N.M.A., van Haperen R., Osterhaus A.D.M.E., van Kuppeveld F.J.M., Haagmans B.L., Grosveld F., Bosch B.-J. (2020). A human monoclonal antibody blocking SARS-CoV-2 infection. Nat. Commun..

[55] Pinto D., Park Y.-J., Beltramello M., Walls A.C., Tortorici M.A., Bianchi S., Jaconi S., Culap K., Zatta F., Marco A.D. (2020). Cross-neutralization of SARS-CoV-2 by a human monoclonal SARS-CoV antibody. Nature.

[56] Qiu M., Shi Y., Guo Z., Chen Z., He R., Chen R., Zhou D., Dai E., Wang X., Si B. (2005). Antibody responses to individual proteins of SARS coronavirus and their neutralization activities. Microbes Infect..

[57] Tang X.-C., Agnihothram S.S., Jiao Y., Stanhope J., Graham R.L., Peterson E.C., Avnir Y., Tallarico A.S.C., Sheehan J., Zhu Q. (2014). Identification of human neutralizing antibodies against MERS-CoV and their role in virus adaptive evolution. Proc. Natl. Acad. Sci. USA.

[58] Siriwattananon K., Manopwisedjaroen S., Shanmugaraj B., Rattanapisit K., Phumiamorn S., Sapsutthipas S., Trisiriwanich S., Prompetchara E., Ketloy C., Buranapraditkun S. (2021). Plant-produced receptor-binding domain of SARS-CoV-2 elicits potent neutralizing responses in mice and non-human primates. Front. Plant Sci..

[59] He Y., Li J., Heck S., Lustigman S., Jiang S. (2006). Antigenic and immunogenic characterization of recombinant baculovirus-expressed severe acute respiratory syndrome coronavirus spike protein: Implication for vaccine design. J. Virol..

[60] Wang Y., Tai W., Yang J., Zhao G., Sun S., Tseng C.-T.K., Jiang S., Zhou Y., Du L., Gao J. (2017). Receptor-binding domain of MERS-CoV with optimal immunogen dosage and immunization interval protects human transgenic mice from MERS-CoV infection. Hum. Vaccines Immunother..

[61] Liu Z., Xu W., Xia S., Gu C., Wang X., Wang Q., Zhou J., Wu Y., Cai X., Qu D. (2020). RBD-Fc-based COVID-19 vaccine candidate induces highly potent SARS-CoV-2 neutralizing antibody response. Signal Transduct. Target. Ther..

[62] Muthumani K., Falzarano D., Reuschel E.L., Tingey C., Flingai S., Villarreal D.O., Wise M., Patel A., Izmirly A., Aljuaid A. (2015). A synthetic consensus anti-spike protein DNA vaccine induces protective immunity against Middle East respiratory syndrome coronavirus in nonhuman primates. Sci. Transl. Med..

[63] Modjarrad K., Roberts C.C., Mills K.T., Castellano A.R., Paolino K., Muthumani K., Reuschel E.L., Robb M.L., Racine T., Oh M.-D. (2019). Safety and immunogenicity of an anti-Middle East respiratory syndrome coronavirus DNA vaccine: A phase 1, open-label, single-arm, dose-escalation trial. Lancet Infect. Dis..

[64] Tai W., Wang Y., Fett C.A., Zhao G., Li F., Perlman S., Jiang S., Zhou Y., Du L. (2016). Recombinant receptor-binding domains of multiple Middle East respiratory syndrome coronaviruses (MERS-CoVs) induce cross-neutralizing antibodies against divergent human and camel MERS-CoVs and antibody escape mutants. J. Virol..

[65] Wu Z., Hu Y., Xu M., Chen Z., Yang W., Jiang Z., Li M., Jin H., Cui G., Chen P. (2021). Safety, tolerability, and immunogenicity of an inactivated SARS-CoV-2 vaccine (CoronaVac) in healthy adults aged 60 years and older: A randomised, double-blind, placebo-controlled, phase 1/2 clinical trial. Lancet Infect. Dis..

[66] Ella R., Vadrevu K.M., Jogdand H., Prasad S., Reddy S., Sarangi V., Ganneru B., Sapkal G., Yadav P., Abraham P. (2021). Safety and immunogenicity of an inactivated SARS-CoV-2 vaccine, BBV152: A double-blind, randomised, phase 1 trial. Lancet Infect. Dis..

[67] Anderson E.J., Rouphael N.G., Widge A.T., Jackson L.A., Roberts P.C., Makhene M., Chappell J.D., Denison M.R., Stevens L.J., Pruijssers A.J. (2020). Safety and immunogenicity of SARS-CoV-2 mRNA-1273 vaccine in older adults. N. Engl. J. Med..

[68] Walsh E.E., Frenck R.W., Falsey A.R., Kitchin N., Absalon J., Gurtman A., Lockhart S., Neuzil K., Mulligan M.J., Bailey R. (2020). Safety and immunogenicity of two RNA-based COVID-19 vaccine candidates. N. Engl. J. Med..

[69] Folegatti P.M., Ewer K.J., Aley P.K., Angus B., Becker S., Belij-Rammerstorfer S., Bellamy D., Bibi S., Bittaye M., Clutterbuck E.A. (2020). Safety and immunogenicity of the ChAdOx1 nCoV-19 vaccine against SARS-CoV-2: A preliminary report of a phase 1/2, single-blind, randomised controlled trial. Lancet.

[70] Novavax Novavax COVID-19 Vaccine Demonstrates 89.3% Efficacy in UK Phase 3 Trial. https://ir.novavax.com/news-releases/news-release-details/novavax-covid-19-vaccine-demonstrates-893-efficacy-uk-phase-3.

[71] Pavlou A.K., Reichert J.M. (2004). Recombinant protein therapeutics - success rates, market trends and values to 2010. Nat. Biotechnol..

[72] Barta A., Sommergruber K., Thompson D., Hartmuth K., Matzke M.A., Matzke A.J. (1986). The expression of a nopaline synthase—Human growth hormone chimaeric gene in transformed tobacco and sunflower callus tissue. Plant Mol. Biol..

[73] Mor T.S. (2015). Molecular pharming’s foot in the FDA’s door: Protalix’s trailblazing story. Biotechnol. Lett..

[74] Shanmugaraj B., Bulaon C.J.I., Malla A., Phoolcharoen W. (2021). Biotechnological insights on the expression and production of antimicrobial peptides in plants. Molecules.

[75] Ma J.K.-C., Drossard J., Lewis D., Altmann F., Boyle J., Christou P., Cole T., Dale P., van Dolleweerd C.J., Isitt V. (2015). Regulatory approval and a first-in-human phase I clinical trial of a monoclonal antibody produced in transgenic tobacco plants. Plant Biotechnol. J..

[76] van Dolleweerd C.J., Teh A.Y.-H., Banyard A.C., Both L., Lotter-Stark H.C.T., Tsekoa T., Phahladira B., Shumba W., Chakauya E., Sabeta C.T. (2014). Engineering, Expression in Transgenic Plants and Characterisation of E559, a Rabies Virus-Neutralising Monoclonal Antibody. J. Infect. Dis..

[77] Hendin H.E., Pillet S., Lara A.N., Wu C.-Y., Charland N., Landry N., Ward B.J. (2017). Plant-made virus-like particle vaccines bearing the hemagglutinin of either seasonal (H1) or avian (H5) influenza have distinct patterns of interaction with human immune cells in vitro. Vaccine.

[78] Boonyayothin W., Sinnung S., Shanmugaraj B., Abe Y., Strasser R., Pavasant P., Phoolcharoen W. (2021). Expression and functional evaluation of recombinant anti-receptor activator of nuclear factor kappa-B ligand monoclonal antibody produced in *Nicotiana benthamiana*. Front. Plant Sci..

[79] Mercx S., Smargiasso N., Chaumont F., Pauw E.D., Boutry M., Navarre C. (2017). Inactivation of the β(1,2)-xylosyltransferase and the α(1,3)-fucosyltransferase genes in *Nicotiana tabacum* BY-2 Cells by a Multiplex CRISPR/cas9 strategy results in glycoproteins without plant-specific glycans. Front. Plant Sci..

[80] Kizhner T., Azulay Y., Hainrichson M., Tekoah Y., Arvatz G., Shulman A., Ruderfer I., Aviezer D., Shaaltiela Y. (2015). Characterization of a chemically modified plant cell culture expressed human a-Galactosidase-A enzyme for treatment of Fabry disease. Mol. Genet. Metab..

[81] Rattanapisit K., Shanmugaraj B., Manopwisedjaroen S., Purwono P.B., Siriwattananon K., Khorattanakulchai N., Hanittinan O., Boonyayothin W., Thitithanyanont A., Smith D.R. (2020). Rapid production of SARS-CoV-2 receptor binding domain (RBD) and spike specific monoclonal antibody CR3022 in *Nicotiana benthamiana*. Sci. Rep..

[82] Shanmugaraj B., Phoolcharoen W. (2021). Addressing demand for recombinant biopharmaceuticals in the COVID-19 era. Asian Pac. J. Trop. Med..

[83] Maharjan P.M., Choe S. (2019). Transient expression of hemagglutinin antigen from canine influenza virus H3N2 in *Nicotiana benthamiana* and *Lactuca sativa*. Clin. Exp. Vaccine Res..

[84] Chen Q., Dent M., Hurtado J., Stahnke J., McNulty A., Leuzinger K., Lai H. (2016). Transient Protein Expression by Agroinfiltration in Lettuce. Methods Mol. Biol..

[85] Seki M., Komeda Y., Iida A., Yamada Y., Morikawa H. (1991). Transient expression of beta-glucuronidase in *Arabidopsis thaliana* leaves and roots and *Brassica napus* stems using a pneumatic particle gun. Plant Mol. Biol..

[86] Bernat-Silvestre C., Vieira V.D.S., Sánchez-Simarro J., Aniento F., Marcote M.J. (2021). Transient Transformation of *A. thaliana* Seedlings by Vacuum Infiltration. Methods Mol. Biol..

[87] Gengenbach B.B., Keil L.L., Opdensteinen P., Müschen C.R., Melmer G., Lentzen H., Bührmann J., Buyel J.F. (2019). Comparison of microbial and transient expression (tobacco plants and plant-cell packs) for the production and purification of the anticancer mistletoe lectin viscumin. Biotechnol. Bioeng..

[88] Fard A.B., Nayeri F.D., Anbuhi M.H. (2019). Transient expression of etanercept therapeutic protein in tobacco (*Nicotiana tabacum* L.). Int. J. Biol. Macromol..

[89] Siriwattananon K., Manopwisedjaroen S., Kanjanasirirat P., Purwono P.B., Rattanapisit K., Shanmugaraj B., Smith D.R., Borwornpinyo S., Thitithanyanont A., Phoolcharoen W. (2021). Development of plant-produced recombinant ACE2-Fc fusion protein as a potential therapeutic agent against SARS-CoV-2. Front. Plant Sci..

[90] Rattanapisit K., Srifa S., Kaewpungsup P., Pavasant P., Phoolcharoen W. (2019). Plant-produced recombinant Osteopontin-Fc fusion protein enhanced osteogenesis. Biotechnol. Rep..

[91] Rattanapisit K., Chao Z., Siriwattananon K., Huang Z., Phoolcharoen W. (2019). Plant-produced anti-enterovirus 71 (EV71) monoclonal antibody efficiently protects mice against EV71 infection. Plants.

[92] Rattanapisit K., Phakham T., Buranapraditkun S., Siriwattananon K., Boonkrai C., Pisitkun T., Hirankarn N., Strasser R., Abe Y., Phoolcharoen W. (2019). Structural and in vitro functional analyses of novel plant-produced anti-Human PD1 antibody. Sci. Rep..

[93] Pogue G.P., Vojdani F., Palmer K.E., Hiatt E., Hume S., Phelps J., Long L., Bohorova N., Kim D., Pauly M. (2010). Production of pharmaceutical-grade recombinant aprotinin and a monoclonal antibody product using plant-based transient expression systems. Plant Biotechnol. J..

[94] Krenek P., Samajova O., Luptovciak I., Doskocilova A., Komis G., Samaj J. (2015). Transient plant transformation mediated by *Agrobacterium tumefaciens*: Principles, methods and applications. Biotechnol. Adv..

[95] Komarova T.V., Baschieri S., Donini M., Marusic C., Benvenuto E., Dorokhov Y.L. (2010). Transient expression systems for plant-derived biopharmaceuticals. Expert Rev. Vaccines.

[96] Kim M.J., Baek K., Park C.-M. (2009). Optimization of conditions for transient Agrobacterium-mediated gene expression assays in *Arabidopsis*. Plant Cell Rep..

[97] Lee M.W., Yang Y. (2006). Transient expression assay by agroinfiltration of leaves. Methods Mol. Biol..

[98] Wigdorovitz A., Filgueira D.M.P., Robertson N., Carrillo C., Sadir A.M., Morris T.J., Borca M.V. (1999). Protection of mice against challenge with foot and mouth disease virus (FMDV) by immunization with foliar extracts from plants infected with recombinant tobacco mosaic virus expressing the FMDV structural protein VP1. Virology.

[99] Santi L., Batchelor L., Huang Z., Hjelm B., Kilbourne J., Arntzen C.J., Chen Q., Mason H.S. (2008). An efficient plant viral expression system generating orally immunogenic Norwalk virus-like particles. Vaccine.

[100] Phoolcharoen W., Bhoo S.H., Lai H., Ma J., Arntzen C.J., Chen Q., Mason H.S. (2011). Expression of an immunogenic Ebola immune complex in *Nicotiana benthamiana*. Plant Biotechnol. J..

[101] Gómez M.L., Huang X., Alvarez D., He W., Baysal C., Zhu C., Armario-Najera V., Perera A.B., Bennasser P.C., Saba-Mayoral A. (2021). Contributions of the international plant science community to the fight against human infectious diseases—Part 1: Epidemic and pandemic diseases. Plant Biotechnol. J..

[102] He W., Baysal C., Gómez M.L., Huang X., Alvarez D., Zhu C., Armario-Najera V., Perera A.B., Bennaser P.C., Saba-Mayoral A. (2021). Contributions of the international plant science community to the fight against infectious diseases in humans—Part 2: Affordable drugs in edible plants for endemic and re-emerging diseases. Plant Biotechnol. J..

[103] Sainsbury F. (2020). Innovation in plant-based transient protein expression for infectious disease prevention and preparedness. Curr. Opin. Biotechnol..

[104] Aziz M.A., Sikriwal D., Singh S., Jarugula S., Kumar P.A., Bhatnagar R. (2005). Transformation of an edible crop with the pagA gene of Bacillus anthracis. FASEB J..

[105] Lakshmi P.S., Verma D., Yang X., Lloyd B., Daniell H. (2013). Low Cost Tuberculosis Vaccine Antigens in Capsules: Expression in Chloroplasts, Bio-Encapsulation, Stability and Functional Evaluation In Vitro. PLoS ONE.

[106] Pogrebnyak N., Golovkin M., Andrianov V., Spitsin S., Smirnov Y., Egolf R., Koprowski H. (2005). Severe acute respiratory syndrome (SARS) S protein production in plants: Development of recombinant vaccine. Proc. Natl. Acad. Sci. USA.

[107] Zheng N., Xia R., Yang C., Yin B., Li Y., Duan C., Liang L., Guo H., Xie Q. (2009). Boosted expression of the SARS-CoV nucleocapsid protein in tobacco and its immunogenicity in mice. Vaccine.

[108] Faye L., Gomord V. (2010). Success stories in molecular farming-a brief overview. Plant Biotechnol. J..

[109] Pillet S., Aubin É., Trépanier S., Bussière D., Dargis M., Poulin J.-F., Yassine-Diab B., Ward B.J., Landry N. (2016). A plant-derived quadrivalent virus like particle influenza vaccine induces cross-reactive antibody and T cell response in healthy adults. Clin. Immunol..

[110] Ward B.J., Makarkov A., Séguin A., Pillet S., Trépanier S., Dhaliwall J., Libman M.D., Vesikari T., Landry N. (2020). Efficacy, immunogenicity, and safety of a plant-derived, quadrivalent, virus-like particle influenza vaccine in adults (18–64 years) and older adults (≥65 years): Two multicentre, randomised phase 3 trials. Lancet.

[111] Márquez-Escobar V.A., Rosales-Mendoza S., Beltrán-López J.I., González-Ortega O. (2017). Plant-based vaccines against respiratory diseases: Current status and future prospects. Expert Rev. Vaccines.

[112] Li H.-Y., Ramalingam S., Chye M.-L. (2006). Accumulation of recombinant SARS-CoV spike protein in plant cytosol and chloroplasts indicate potential for development of plant-derived oral vaccines. Exp. Biol. Med..

[113] Demurtas O.C., Massa S., Illiano E., Martinis D.D., Chan P.K.S., Bonito P.D., Franconi R. (2016). Antigen production in plant to tackle infectious diseases flare up: The case of SARS. Front. Plant Sci..

[114] Diego-Martin B., González B., Vazquez-Vilar M., Selma S., Mateos-Fernández R., Gianoglio S., Fernández-del-Carmen A., Orzáez D. (2020). Pilot production of SARS-CoV-2 related proteins in plants: A proof of concept for rapid repurposing of indoor farms into biomanufacturing facilities. Front. Plant Sci..

[115] British American Tobacco BAT Makes Progress on COVID-19 Vaccine & Provides Community Support. https://www.bat.com/group/sites/UK__9D9KCY.nsf/vwPagesWebLive/DOBPMBZC#.

[116] iBio IBIO-201 Demonstrates Ability to Elicit Anti-SARS-CoV-2 Immune Response in Preclinical Studies. https://www.ibioinc.com/ibio-provides-update-on-ibio-201-covid-19-vaccine-program/.

[117] iBio iBio Reports Successful Preclinical Immunization Studies with Next-Gen Nucleocapsid COVID-19 Vaccine Candidate. https://ibioinc.com/ibio-reports-successful-preclinical-immunization-studies-with-next-gen-nucleocapsid-covid-19-vaccine-candidate/.

[118] The Canadian Press Medicago Reports Promising but Preliminary Results of Possible COVID-19 Vaccine. https://www.cbc.ca/news/health/medicago-covid-19-vaccine-phase-1-1.5796411.

[119] Kumar A.U., Kadiresen K., Gan W.C., Ling A.P.K. (2021). Current updates and research on plant-based vaccines for coronavirus disease 2019. Clin. Exp. Vaccine Res..

[120] Ward B.J., Gobeil P., Séguin A., Atkins J., Boulay I., Charbonneau P.-Y., Couture M., D’Aoust M.-A., Dhaliwall J., Finkle C. (2021). Phase 1 randomized trial of a plant-derived virus-like particle vaccine for COVID-19. Nat. Med..

[121] Pillet S., Arunachalam P.S., Andreani G., Golden N., Fontenot J., Aye P., Röltgen K., Lehmick G., Dubé C., Gobeil P. (2021). Safety, immunogenicity and protection provided by unadjuvanted and adjuvanted formulations of recombinant plant-derived virus-like particle vaccine candidate for COVID-19 in non-human primates. bioRxiv.

[122] Dai L., Gao G.F. (2021). Viral targets for vaccines against COVID-19. Nat. Rev. Immunol..

[123] Dutta N.K., Mazumdar K., Gordy J.T. (2020). The Nucleocapsid Protein of SARS-CoV-2: A Target for Vaccine Development. J. Virol..

[124] Oliveira S.C., de Magalhães M.T.Q., Homan E.J. (2020). Immunoinformatic Analysis of SARS-CoV-2 Nucleocapsid Protein and Identification of COVID-19 Vaccine Targets. Front. Immunol..

[125] Zhao P., Cao J., Zhao L.J., Qin Z.L., Ke J.S., Pan W., Ren H., Yu J.G., Qi Z.T. (2005). Immune responses against SARS-coronavirus nucleocapsid protein induced by DNA vaccine. Virology.

[126] Baiya Phytopharm COVID-19 Vaccine Development. https://baiyaphytopharm.com/covid-19/.

[127] Lai H., Engle M., Fuchs A., Keller T., Johnson S., Gorlatov S., Diamond M.S., Chen Q. (2010). Monoclonal antibody produced in plants efficiently treats West Nile virus infection in mice. Proc. Natl. Acad. Sci. USA.

[128] Sainsbury F., Lomonossoff G.P. (2008). Extremely high-level and rapid transient protein production in plants without the use of viral replication. Plant Physiol..

[129] Makatsa M.S., Tincho M.B., Wendoh J.M., Ismail S.D., Nesamari R., Pera F., de Beer S., David A., Jugwanth S., Gededzha M.P. (2021). SARS-CoV-2 Antigens Expressed in Plants Detect Antibody Responses in COVID-19 Patients. Front. Plant Sci..

[130] Rattanapisit K., Yusakul G., Shanmugaraj B., Kittirotruji K., Suwatsrisakul P., Prompetchara E., Taychakhoonavud S., Phoolcharoen W. (2021). Plant-produced recombinant SARS-CoV-2 receptor-binding domain; an economical, scalable biomaterial source for COVID-19 diagnosis. Biomater. Transl..

[131] iBio Therapeutic & Vaccine Candidates. https://ibioinc.com/pipeline/.

[132] Capell T., Twyman R.M., Armario-Najera V., Ma J.K.-C., Schillberg S., Christou P. (2020). Potential applications of plant biotechnology against SARS-CoV-2. Trends Plant Sci..

[133] Leaf Expression Systems COVID-19: What We’re Doing. https://www.leafexpressionsystems.com/covid-19/.

[134] Siriwattananon K., Manopwisedjaroen S., Shanmugaraj B., Prompetchara E., Ketloy C., Buranapraditkun S., Tharakhet K., Kaewpang P., Ruxrungtham K., Thitithanyanont A. (2021). Immunogenicity Studies of Plant-Produced SARS-CoV-2 Receptor Binding Domain-Based Subunit Vaccine Candidate with Different Adjuvant Formulations. Vaccines.

[135] Montero-Morales L., Steinkellner H. (2018). Advanced Plant-Based Glycan Engineering. Front. Bioeng. Biotechnol..

